# Adeno-Associated Virus 5 Protein Particles Produced by *E. coli* Cell-Free Protein Synthesis

**DOI:** 10.1021/acssynbio.4c00403

**Published:** 2024-08-23

**Authors:** Danielle Deuker, Ernest Asilonu, Daniel G. Bracewell, Stefanie Frank

**Affiliations:** † Department of Biochemical Engineering, University College London, Bernard Katz Building, Gower Street, London, WC1E 6BT, United Kingdom; ‡ Cytiva Europe Limited, 5 Harbourgate Business Park, Southampton Road, Portsmouth, Hampshire PO6 4BQ, United Kingdom

**Keywords:** cell-free protein synthesis, AAV, VLP, self-assembling, *in vitro*, *E. coli*

## Abstract

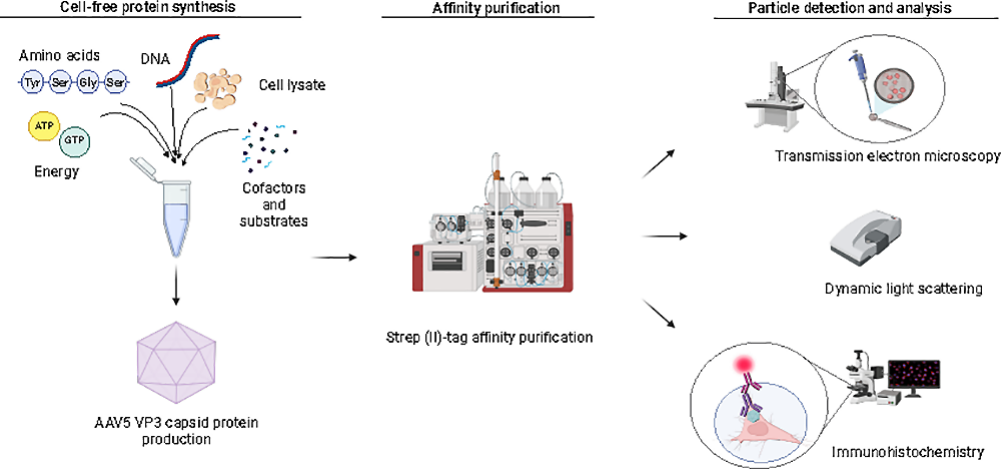

Recombinant adeno-associated viruses (rAAVs) have emerged as important tools for gene therapy and, more recently, vaccine development. Nonetheless, manufacturing can be costly and time-consuming, emphasizing the importance of alternative production platforms. We investigate the potential of *E. coli*-based cell-free protein synthesis (CFPS) to produce recombinant AAV5 virus-like particles (VLPs). AAV5 virus protein 3 (VP3) constructs, both with and without Strep-tag II, were expressed with CFPS. Lower reaction temperatures resulted in increased solubility, with the untagged variant containing nearly 90% more soluble VLP VP3 protein at 18 °C than at 37 °C. Affinity chromatography of N-terminally Strep(II)-tagged VP3 enabled successful isolation with minimal processing. DLS and TEM confirmed the presence of ∼20 nm particles. Furthermore, the N-terminally tagged AAV5 VP3 VLPs were biologically active, successfully internalizing into HeLa cells. This study describes an innovative approach to AAV VLP production using *E. coli*-based CFPS, demonstrating its potential for rapid and biologically active AAV VLP synthesis.

## Introduction

Virus-like particles (VLPs) are noninfectious polymeric nanoparticles formed from virus-coat proteins but lacking genetic material. They have been successfully utilized in various applications including vaccines,^1,2^ gene therapy,^3,4^ drug delivery,^5^ and material science.^6^ VLPs can be produced by various expression hosts including bacteria,^7,8^ mammalian,^9,10^ yeast,^11^ and insect cell systems.^12,13^ Additionally, VLPs, including MS2 and HBcAg,^14−16^ have been produced *in vitro* using cell-free protein synthesis (CFPS), which allows for comparatively rapid synthesis.

**Figure 1:**
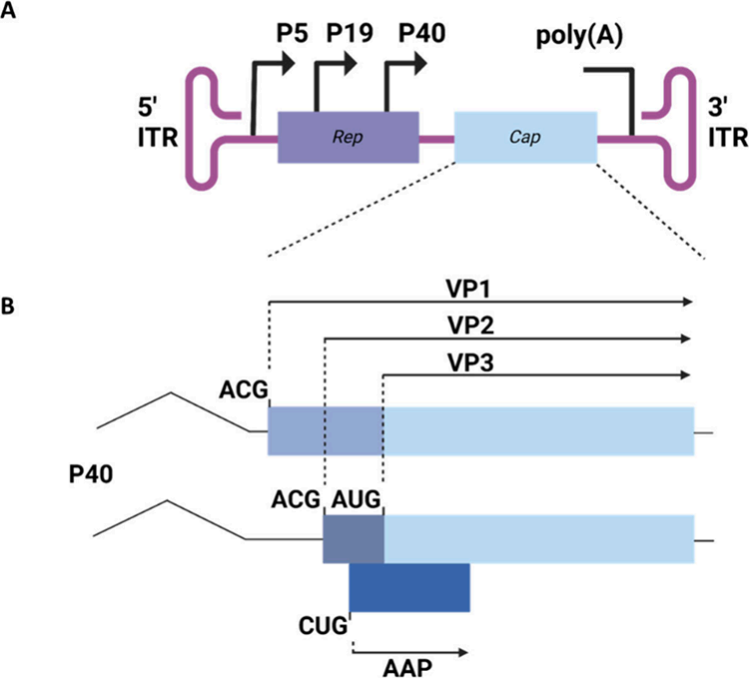
Schematic showing the AAV genome and Cap gene. (A) AAV genome flanked by inverted terminal repeats. The genome contains the Rep (purple) and Cap (blue) genes. The genome is controlled by three different promoters: P5, P19, and P40. (B) Cap gene controlled by the P40 promoter. This gene encodes for 3 capsid proteins, VP1, VP2, and VP3, as a result of alternative mRNA splicing and leaky scanning. VP1, VP2, and VP3 share a C-terminal region (light blue) and differ in their N-termini (gray/blue). For the spliced VP2 and VP3 gene, a weak ACG start codon controls VP2 production and a stronger AUG start codon controls VP3.^17^ AAP (dark blue) is also produced under the P40 promoter on a separate open reading frame and aids capsid assembly.

Adeno-associated viruses (AAV) are nonenveloped single-stranded DNA viruses ranging from 20 to 25 nm in diameter and belonging to the Parvoviridae family. The AAV genome consists of two open reading frames: Rep (replication) and Cap (capsid). The Cap cassette is responsible for encoding structural capsid proteins VP1, VP2, and VP3 (Figure 1), most commonly in a 1:1:10 ratio.^17^ VP3, the primary structural protein, can self-assemble into capsids without the other viral capsid proteins VP1 and VP2.^18^ Further, the Cap gene encodes for assembly activating protein (AAP), which promotes capsid assembly.^19^ However, among 13 naturally occurring AAV serotypes, AAV serotype 5 stands out as the most genetically diverse, displaying the unique ability to assemble capsids in the absence of AAP.^20^

AAV shows much potential within the biomedical field due to its low immunogenicity and lack of pathogenicity.^17,21^ Recombinant AAV (rAAV) vectors have gained prominence within the gene therapy space, with multiple approved drugs utilizing AAV including Zolgensma,^22^ Roctavian, and Hemgenix^23^ and others in clinical trials.^22^ Furthermore, rAAV has shown potential as a promising vaccine candidate.^24,25^ This is because different AAV serotypes can tolerate mutations^17^ for antigen display and can target specific tissues dependent on serotype.^21^ Currently, AAV production is primarily achieved using either HEK-293 cells or Sf9 insect cells.^26^ AAV VLPs have also been produced using other mammalian cell lines and, more recently, *E. coli*.^7,8^ However, these methods of production have their drawbacks including lengthy production times, high costs, low titer, and process-related impurities.^27^

Cell-free protein synthesis offers a promising alternative to cell-based protein production.^28^ By utilizing the cellular machinery *in vitro,* protein production can be achieved rapidly, without the need for laborious workflows associated with cell culturing and at scales ranging from <10 μL to 100 L.^29^ Furthermore, since the system is open in nature, it can be easily manipulated to suit the protein of interest by introducing enzymes and post-translational modifications (PTMs) that may not be possible in prokaryotic cell-based systems.^14,30−32^

This study demonstrated the application of *E. coli*-based CFPS to generate rAAV5 VP3 VLPs. The protein produced is mostly in a soluble form and can spontaneously form capsid particles. Ultimately, we demonstrate that these particles can be taken up by HeLa cells.

## Materials and Methods

### AAV5 VP3 Vector Design and Plasmid Preparation

The AAV5 VP3 sequence was obtained from NCBI (National Center for Biotechnology Information) and codon-optimized using Benchling [Biology Software] (2022); retrieved from https://benchling.com. Three constructs were designed: one with an N-terminal Strep-tag II, one with a C-terminal Strep-tag II, and one without tags. These constructs were cloned into the pJL1 vector using CloneEZ (Genscript). Plasmid pJL1-sfGFP was a gift from Michael Jewett (Addgene plasmid #69496; http://n2t.net/addgene:69496). Sequences for each construct are provided in Supporting Information 1. Synthesized plasmids were amplified followed by purification using QIAGEN plasmid plus maxi kit according to the manufacturer’s instructions.

### *E. coli* Lysate Generation

*E. coli* cell-free lysate was generated using BL21 Star (DE3). An aliquot was thawed on ice and added to 100 mL of LB media. After 16.5 h of incubation at 34 °C, shaking at 250 rpm, this culture was used to inoculate a 500 mL culture to an OD_600_ of 0.05 in 2xYPTG (16 g/L tryptone, 10 g/L yeast extract, 5 g/L sodium chloride, 7 g/L dipotassium phosphate, 4.3 g/L monopotassium phosphate, and 18 g/L glucose, pH 7.2). Once an OD_600_ of 0.6–0.8 was reached, 1 mM IPTG was added for production of T7 RNA polymerase. At OD_600_ = 2, 1 mM KOH was added. At OD_600_ = 4 cells were harvested by centrifugation. Pellets were resuspended in 100 mL of S30 buffer (10 mM Tris-acetate, 21 mM magnesium acetate, 60 mM potassium acetate, 1 mM dithiothreitol), followed by homogenization in a French press homogenizer at 1000 bar pressure. The lysate was centrifuged twice at 30,000 RCF for 30 min. The supernatant was aliquoted, flash frozen with liquid nitrogen, and stored at −80 °C.

### Reaction Buffer

Reaction buffer was based on the PANOx-SP composition first described by Kwon and Jewett.^33^ Reaction buffer was prepared to a 2.5× concentration. A 100 μL CFPS reaction consisted of 1.2 mM ATP, 0.85 mM CTP, 0.85 mM GTP, 0.85 mM UTP, 1.5 mM spermidine, 1 mM putrescince dihydrochloride, 33 mM PEP monosodium salt, 0.27 mM coenzyme A, 0.33 mM NAD, 90 mM potassium glutamate, 10 mM ammonium glutamate, 12 mM magnesium glutamate, 4 mM sodium oxalate, 34 μg/mL folinic acid, and 170 μg/mL *E. coli* tRNA (Roche). Reaction buffer was pH adjusted to pH 7.0 with KOH. The reaction mixture was aliquoted and stored at −80 °C.

Additionally, a concentrated 50 mM amino acid mix was prepared containing l-alanine, l-arginine, l-asparagine, l-aspartic acid, l-cysteine, l-glutamine, l-glutamic acid, glycine, l-histidine, l-isoleucine, l-leucine, l-lysine, l-phenylalanine, l-proline, l-serine, l-threonine, l-tryptophan, l-tyrosine, and l-valine. KOH pellets were added until the amino acids were solubilized according to ref (34). A 75 mM l-methionine solution was also prepared separately.

### Cell-Free Reaction Conditions

100–500 μL CFPS reactions were prepared in 1.5 mL Eppendorf tubes using 20% v/v cell lysate, 40% v/v reaction buffer (described above), 1.25 mM amino acid mixture, 1.5 mM l-methionine, 0.5 U/μL T7 RNA polymerase (New England Biolabs), 1 μg plasmid DNA, and nuclease-free water. Reactions were incubated at 18 °C for 16 h, unless otherwise stated. T7 RNA polymerase was supplemented in addition to the endogenous T7 RNA polymerase present in the cell lysate because literature indicates that this approach increases protein yields.^35^

### Western Blot

A 7.5 μL sample was separated using a 4–12% Bis-Tris gel (Invitrogen). Proteins were transferred from SDS-PAGE to nitrocellulose membranes using the TransBlot Turbo (Bio-Rad) system. Membranes were then blocked in 0.5% w/v nonfat milk in TBST (20 mM Tris, 150 mM NaCl, 0.1% v/v Tween-20) for 1 h at room temperature. After blocking, the milk-TBST solution was replaced with primary antibody anti-AAV VP1/VP2/VP3 mouse monoclonal antibody [clone: B1] (Progen) at dilutions of 1:500 or 1:100. The secondary antibody was alkaline phosphatase-conjugated goat anti-mouse IgG (H+L) at a 1:10,000 dilution. Alternatively, the blots were probed with Strep-Tactin-HRP (IBA). Blots were visualized using BCIP/NBT 1-step substrate (ThermoFisher Scientific) or Supersignal West Pico PLUS chemiluminescent substrate (ThermoFisher Scientific).

### Densitometry

Densitometry analysis was conducted using ImageJ^36^ to relatively quantify soluble and insoluble protein bands. Percentage protein produced under different conditions was determined.

### Affinity Purification

Affinity purification of CFPS products was performed using a 1 mL StrepTrap XT column (Cytiva) on an ÄKTA PURE FPLC system (Cytiva). Four 500 μL (2 mL) CFPS reactions were centrifuged at 4 °C and 12,000 RCF for 2 min, and the supernatant was loaded onto the column. The column was washed with 150 mM NaCL, 100 mM Tris-HCl, and 1 mM EDTA, pH 8.0. Bound material was eluted using 150 mM NaCL, 100 mM Tris-HCl, 1 mM EDTA, and 50 mM biotin, pH 8.0. The column was regenerated with 0.5 M NaOH. Eluted samples were concentrated to 2 mL using Vivaspin centrifugal filters (Cytiva) with a 100 kDa molecular weight cutoff (MWCO).

### ELISA

Purified samples of 50 μL were buffer exchanged 5× into PBS containing 0.01% pluronic-68 acid using Vivaspin 500 centrifugal filters (Sartorius) with a 100 kDa MWCO. Samples were concentrated to a volume of 5 μL and then diluted 1:40 with 1× ASSB buffer (Progen).

ELISA was performed using PROGEN’s AAV5 Xpress ELISA kit (Progen, catalogue no. PRAAV5XP) according to manufacturer’s instructions. Samples and standards were produced in duplicate. The standard curve was fitted using a four-parameter logistic (4PL) model using GraphPad Prism software.^37^

### Dynamic Light Scattering (DLS)

The Zetasizer (Malvern Panalytical) was utilized to conduct DLS using a low-volume quartz cuvette (Malvern Panalytical). A He–Ne laser operating at a wavelength of 633 nm was employed. The instrument configuration was automatically optimized for AAVs using the ZS EXPLORER software (Malvern Panalytical). This configuration utilizes backscatter to detect size. The DLS measurements were conducted three times.

### Transmission Electron Microscopy (TEM)

A 0.2 mg sample was applied onto a Formvar-coated copper grid (Agar) for 5 min. Negative staining was performed by incubating grids in 2% v/v uranyl acetate for 1 min. Excess stain was blotted with a filter paper. Grids were briefly washed with deionized H_2_O before drying. Dried grids were captured on the JEOL 1010 electron microscope with a Gatan Rio camera, and images were analyzed using ImageJ.^36^

### AAV Internalization Study

HeLa cells were seeded in six-well plates containing l-lysine-coated coverslips (Neuvito Corporation) at a density of 1.2e^6^ cells per well and incubated overnight at 37 °C, 5% CO_2_. When cells reached ∼75% confluency, they were incubated with either 4.1e^11^ empty capsid particles per well, an equivalent protein concentration of AAV5 VP3 N-terminal Strep-tag II as determined by bicinchoninic acid (BCA) assay (Pierce), or an equivalent volume of PBS. Samples were incubated at 37 °C with 5% CO_2_ for 2.5 h. Cells were washed with PBS before fixing and permeabilizing with 4% v/v formaldehyde and 0.4% v/v Triton-X 100, respectively. Following this, cells were blocked with 2% BSA in PBS and incubated with ADK5a antibody (Progen) at 4 °C overnight. A secondary antibody fluorescently labeled with Dylight 649 (Rockland) was used as the detection antibody. Cells underwent a 10 min incubation with 4′,6-diamidino-2-phenylindole (DAPI). Subsequently, the coverslips were fixed to glass slides and imaged using a ZEISS LSM 980 confocal microscope. Wavelengths of 465 and 353 nm (DAPI) or 668 and 653 nm (Dylight649) were used to view fluorescence. Image analysis was performed using FIJI.^38^

## Results and Discussion

### AAV5 VP3 Protein Successfully Expressed in the *E. coli* CFPS System

**Figure 2:**
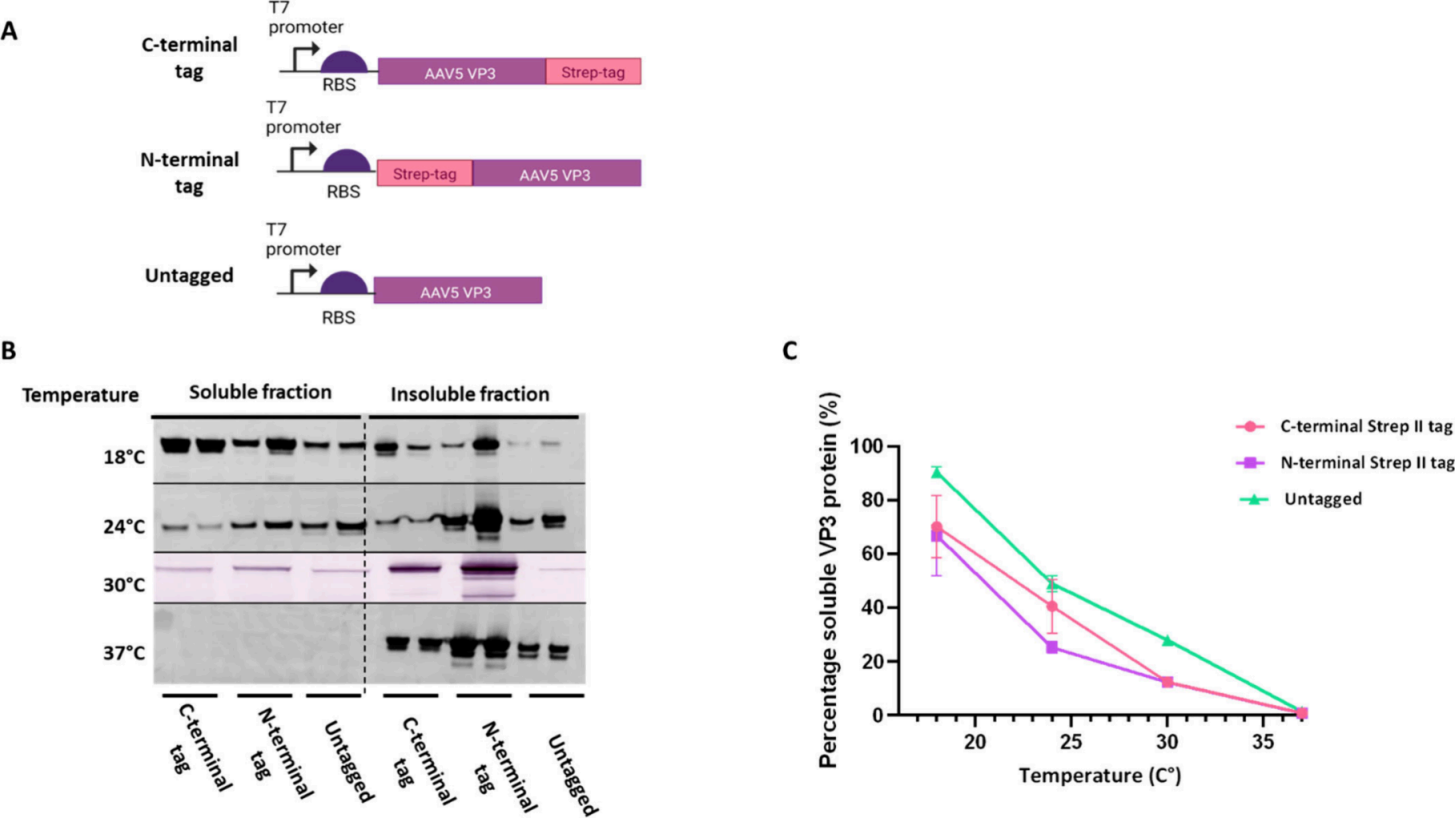
Expression of AAV5 VP3 using CFPS. (A) AAV5 VP3 gene designs that were cloned into the pJL1 vector. (B) Western blot analysis of VP3 protein detected with the B1 antibody from reactions incubated at different temperatures. CFPS reactions were clarified at the end of the reaction, and soluble and insoluble fractions were analyzed. Biological repeats are shown next to each other with the exception of the 30 °C condition, in which two biological repeats were combined for the Western blot. (C) Densitometry analysis of Western blot bands shown in part B. Biological repeats are plotted as a mean with standard deviation shown in the error bars. Band intensity is shown as an average percentage of VP3 soluble protein produced from temperature conditions.

Previous research demonstrated that VP3 protein, expressed and purified from *E. coli*, has the capability to self-assemble into AAV VP3 capsids under controlled *in vitro* conditions.^7,8^ These studies have served as a proof of concept for a novel expression system.^7,8^ In this study, an open CFPS system is described that allows simple expression, assembly, and purification of AAV5 VP3 protein. Since we utilize a CFPS system containing an *E. coli* lysate, the AAV5 VP3 sequence was optimized for *E. coli* codon usage before cloning the gene into the pJL1 vector, which was optimized by others for cell-free synthesis by simplifying the plasmid pY71.^39^ A further two constructs containing a Strep-tag II at either the N- or C-terminus were also cloned into the same vector (Figure 2A).

Protein produced from 16 h CFPS reactions was identified with Western blot using primary antibody B1, which detects unassembled monomeric AAV capsid protein.^8^ VP3 was observed at a molecular weight of 60 kDa, as expected (Figure S1).

To investigate the impact of temperature on production of soluble protein, four reaction temperatures (18, 24, 30, and 37 °C) were investigated and visualized by Western blot (Figure 2B). VP3 was detected at all temperatures; however, at lower temperatures more protein was seen in the soluble fractions. Densitometry analysis of Western blot bands showed a 90% increase in soluble VLP protein for the untagged variant produced at 18 °C compared to 37 °C. Both tagged variants showed an over 60% increase in soluble VP3 protein (Figure 2C). This may be due to increased protein misfolding at higher temperatures due to an increase in temperature-dependent hydrophobic interactions, leading to aggregation.^40^ Multiple smaller bands (Figure S1) were also observed, indicating unstable production or protease activity. This could also be the result of translation initiation at an alternative site within the gene. However, it is unlikely to be due to early termination of translation, as the B1 antibody recognizes an epitope on the C-terminal end of the capsid protein.^8^ Similar observations were reported when using *E. coli*-based AAV protein synthesis. Le *et al.* produced soluble AAV5 VP3 when culturing *E. coli* at 18 °C but detected multiple smaller protein fragments in Western blot analysis,^8^ likely due to protease activity. Cell-free reactions containing no plasmid DNA (Figure S1) showed some nonspecific background signal, which could explain the presence of some of these smaller bands.

### Affinity Purification and Characterization of AAV5 VP3 Capsid Particles

A StrepTrap XT (Cytiva) column was used to purify the Strep-tag II constructs from CFPS reactions. Attempts to purify C-terminally tagged VP3 were unsuccessful (data not shown) potentially due to the tag being obscured. However, protein structure prediction of the VP3 with Strep-tag II was unable to confidently predict the tag position.

**Figure 3:**
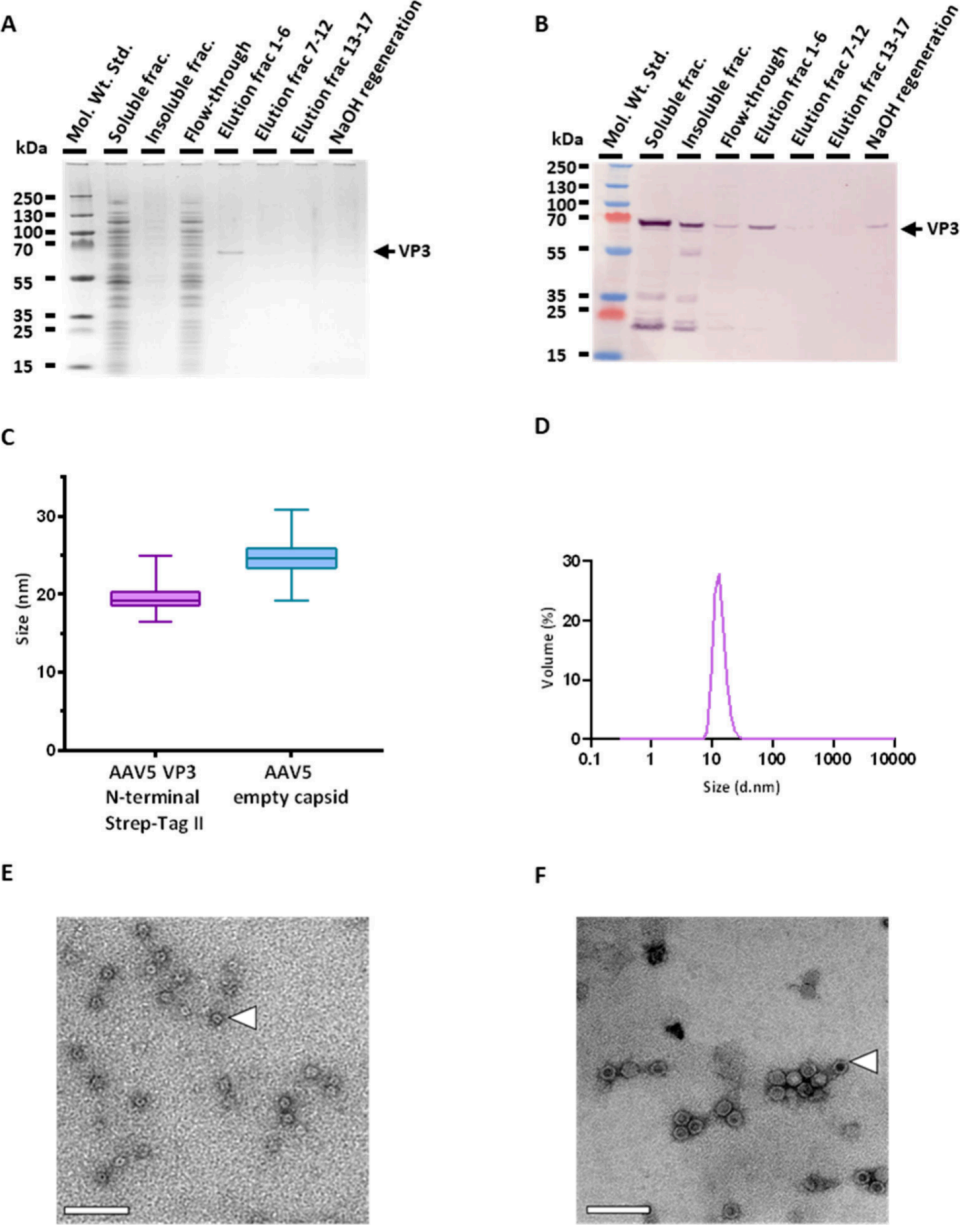
StrepTrapXT affinity chromatography and subsequent particle analysis of N-terminal Strep-tag II AAV5 VP3 capsid particles. (A) Coomassie stain of different samples obtained in purification. (B) B1 Western blot of the same samples used in A. (C) Box and violin plot showing size analysis data derived from TEM images for empty AAV5 particles compared to CFPS-generated AAV5 VP3 particles. The central line indicates the median value, and upper and lower limits of the box indicate the 75% and 25% quartiles. (D) DLS analysis of AAV5 VP3 N-terminal Strep-tag II purified by affinity chromatography. Lines represent an automatically generated average of 3 readings by the ZetaSizer (Malvern Technologies). Peak is shown by volume and measures 13.1 d. nm. (E) TEM analysis of purified VP3 particles. White arrow indicates an empty VP3 VLP particle. (F) TEM analysis of commercial AAV5 capsid control. White arrow indicates an empty AAV5 capsid. Scale bars in (E) and (F) represent 100 nm.

Soluble N-terminally tagged VP3 protein was successfully isolated from StrepTrapXT resin directly from the soluble fraction of a crude CFPS reaction (Figure 3A and 3B). Although VP3 purified using this method appears pure by Coomassie SDS-PAGE staining, a Western blot using a Strep-Tactin HRP conjugate (IBA) on the same samples (Figure 3B), as opposed to the B1 antibody, reveals a protein band at 21 kDa that elutes alongside the protein of interest (Figure S2). This is likely to be a biotinylated protein produced by *E. coli*, which competes with Strep-tag II on the column, as biotin has a stronger affinity to the resin than a Strep-tag II and will displace more weakly bound proteins.^41^ Western blots of samples obtained during the purification procedure also indicate some VP3 is not binding to the resin (Figure 3B). This may be because the biotinylated protein impurity has a stronger affinity to the resin than the Strep-tag II, thus hindering full binding of VP3 proteins. Additionally, further AAV product can be observed within the “NaOH regeneration” fractions, which were collected when stripping the column with NaOH, indicating protein may be aggregating on the column. Despite this, most of the desirable protein can be observed in the initial fractions of the elution step (Figure 3B).

Purifying the AAV VP3 samples allowed for investigation of capsid protein particle assembly. A Progen AAV5 Xpress ELISA, utilizing the ADK5a antibody (Progen), which detects assembled AAV5 particles, was used to determine an estimated particle titer of 2.98e^9^ capsids/mL from the purified sample (Figure S3). This is equivalent to an estimated production yield of 2.98e^9^ capsids/mL of cell-free reagent. DLS was used to explore particle sizes within the purified sample. Three peaks were observed, one at 20.23 diameter in nanometers (d. nm), which is within the expected range of an AAV5 particle^8^ (Figure S4), and two larger peaks at 108 and 5468 d. nm, which may be a result of aggregates. Larger contaminants cause a high intensity of scatter but may not be representative of particle size distribution within the sample, as can be seen when presenting the scatter as “volume” (Figure 3D) or “number” (Figure S4).^42^ Scatter intensities for “volume” and “number” were calculated from the “intensity” plot. The commercial empty AAV5 capsid control (Promega) showed similar sizing by DLS to the CFPS-produced VLP (Figure S4).

Since DLS analysis is disproportionately influenced by larger particles and can be variable,^42^ TEM was used to further observe particle formation and size (Figure 3E). TEM particle analysis revealed N-terminal Strep-tag II VLPs with an average diameter of 19.6 ± 1.8 nm based on analysis of 80 particles, whereas empty AAV5 capsid control particles (Progen) measured 24.6 ± 2.0 nm (Figure 3C,F). This size discrepancy may be due to particles being formed of only VP3, the smallest capsid protein, whereas the control capsids are composed of VP1, VP2, and VP3. Larger unknown artifacts were excluded from this analysis (Figure S5).

### AAV5 VP3 Internalization into HeLa Cells

**Figure 4:**
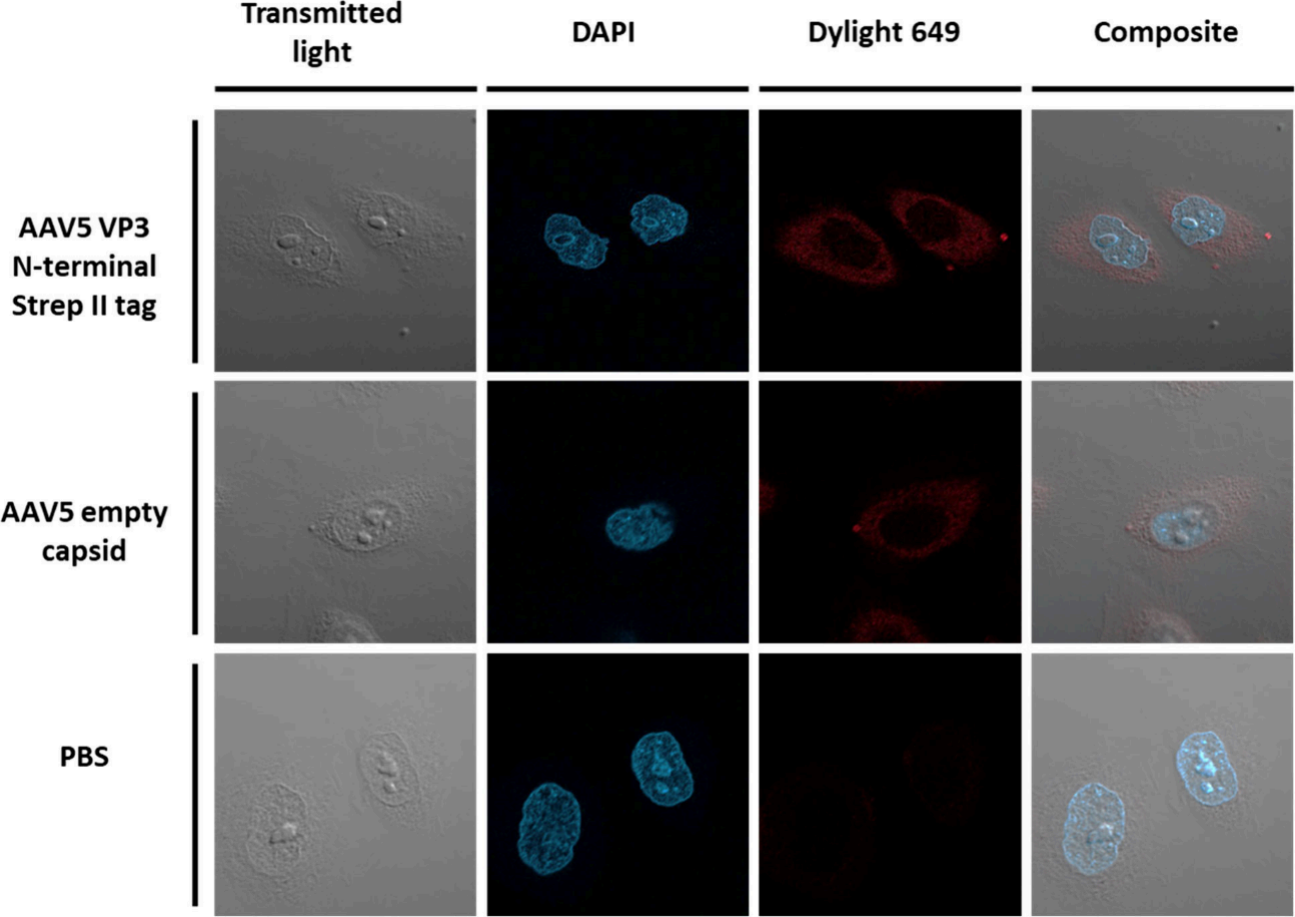
Fluorescence microscopy images of HeLa cells incubated with either AAV5 VP3 N-terminal Strep-tag II (CFPS), AAV5 empty capsid (positive control), or PBS. Cells were incubated with AdK5a followed by a secondary antibody conjugated to Dylight649. The cell nucleus was also stained using DAPI. The cell boundaries were visualized using transmitted light. Images were collected with a 63 x objective.

AAV5 cell entry is driven by receptor-mediated endocytosis. This is achieved when AAV5 particles initially bind to sialic acid residues on the host cell surface, followed by interactions with the polycystic kidney disease 1 (PDK1) domain of the host cell-derived AAV receptor (AAVR), allowing for receptor-mediated endocytosis.^43^ Since the capsid surface is formed by AAV VP3 regions, including protrusions at the 3-fold axis that mediate cell receptor attachment,^44^ VP3-only AAV particles can integrate into cells.^8^ However, VP1 and VP2 are required for nuclear localization and entry.^45^ Internalization of our VP3 VLPs was assessed to investigate their biological activity. HeLa cells, which allow AAV5 propagation,^46^ were fixed to glass slides before permeabilizing and incubating with antibody AdK5a (Progen).^47^ Subsequently, cells were stained with a secondary antibody conjugated with Dylight649 fluorescent dye (red) and DAPI (blue). Transmitted light highlighted the cell boundaries. Negative controls showed minimal red fluorescence signal, whereas for the positive capsid control (empty AAV5, Progen) and the CFPS-derived AAV5 VP3 VLP, red signal can be seen throughout the cell’s cytoplasm (Figure 4). The similar staining profile between the CFPS-derived AAV5 VP3 and the capsid control suggests that the CFPS-created VP3 VLPs can enter HeLa cells. This shows that CFPS-produced AAV5 VP3 VLPs have the appropriate morphology for receptor-mediated endocytosis. Since the CFPS-derived capsids contain only VP3, they would be unable to translocate to the nucleus; hence, we would not expect any signal. Lack of signal in the nucleus for the positive control samples can be explained by the short incubation time; additionally, evidence suggests AAV5 capsids dissemble before the genome enters the nucleus.^46^

## Conclusion

Here we present a novel alternative method for AAV VLP production using *E. coli* based CFPS, that can be used to create pure AAV5 VP3 VLP particles in only 24 h. This method foregoes time-consuming and laborious cell culture steps needed for mammalian and microbial cell culture. Soluble AAV5 VP3 protein production is followed by a short centrifugation step before affinity purification. This process is comparatively easier than the steps of resuspension, high-pressure homogenization, and precipitation required for purifying AAV5 VLP protein from *E. coli* cell culture, as demonstrated previously.^8^ In CFPS, protein was largely produced in soluble form, allowing for simple purification. Previously, AAV2 VP2 particles with varying levels of N-terminal truncation fused with a His_6_ tag were isolated using nickel affinity chromatography, suggesting the tag was exposed on the capsid surface.^48^ Our study is the first report of AAV5 VP3 purified from an N-terminal Strep-tag II. Given the growing interest in the use of AAV VLPs for vaccine development, our findings may be informative for N-terminal antigen display design. Further, we provide evidence of capsid formation and cell internalization of the N-terminal Strep-tag II variant. Nevertheless, there are still hurdles to overcome. Protein yields fluctuate among biological replicates, and purified capsid titers are relatively low. Subsequent efforts will be directed toward resolving these challenges through refined purification techniques and modifications to the cell-free system.

Further, CFPS provides an exciting and open platform that can allow for the inclusion of PTMs. This is beneficial for AAVs, as they exhibit a range of PTMs including acetylation, glycosylation, SUMOlation, and phosphorylation.^49^ It has also been shown that altering PTMs can enhance tissue tropisms.^50^ Moreover, CFPS allows for easy incorporation of cofactors and additives to improve yield and capsid assembly. While there is room for process improvement, this unique method of producing AAVs could lay the foundations for a rapid alternative for producing AAV VLPs with the potential for improved yield and inclusion of additional capsid proteins. This method also provides promise for cargo loading, and since AAV capsids are assembled prior to gene loading, empty rAAVs could allow *in vitro* gene packaging after capsid assembly.^51^ Furthermore, the rapid nature and flexibility of the system could prove to be an exciting, high-throughput screening tool for alternative rAAV serotypes and mutants. This approach is also applicable to the expanding field of engineering modified VLPs, viruses, and other self-assembling particles, as it facilitates rapid prototyping for these complex protein products.^16,52−54^ Overall, cell-free protein synthesis significantly benefits upstream processing in biomanufacturing and research contexts.

## Supporting Information Available

Plasmid and DNA sequences used in this study, full rAAV5 VP3 expression Western blots (Figure S1), Western blot of purification products probed for Strep-Tactin (Figure S2), ELISA standard curve (Figure S3), DLS number and volume distributions (Figure S4), representative TEM image of purified rAAV5 VP3 Strep-tag II (Figure S5) (PDF)

## Abbreviations

rAAV: recombinant adeno-associated virus
VP: virus protein
BSA: bovine serum albumin
CFPS: cell-free protein synthesis
DAPI: 4′,6-diamidino-2-phenylindole
DLS: dynamic light scattering
ELISA: enzyme-linked immunosorbent assay
IPTG: isopropyl β-d-thiogalactopyranoside
PANOx-SP: phosphoenolpyruvate (PEP), amino acids, NAD^+^, and oxalic acid
PBS: phosphate-buffered saline
SDS-PAGE: sodium dodecyl sulfate polyacrylamide gel electrophoresis
TBS: tris-buffered saline
TBST: tris-buffered saline Tween
TEM: transmission electron microscopy
VLPs: virus-like particles
