# Supplementary material for: Adeno-Associated Virus 5 Protein Particles Produced by E. coli Cell-Free Protein Synthesis
Source: ACS Synth Biol. 2024 Aug 23;13(9):2710–7. doi: 10.1021/acssynbio.4c00403 (PMC11421080; doi:10.1021/acssynbio.4c00403)
Supplement: Supplementary file 1 — sb4c00403_si_001.pdf [file sb4c00403_si_001.pdf]

Supporting information

Adeno-Associated Virus 5 Protein Particles produced by *E. coli* Cell-Free Protein Synthesis

Danielle Deuker[1], Ernest Asilonu[2], Daniel Bracewell[1] and Stefanie Frank[1]*

[1] Department of Biochemical Engineering, University College London, Bernard Katz Building, Gower Street, London, WC1E 6BT, United Kingdom

[2] Cytiva Europe Limited, 5 Harbourgate Business Park, Southampton Road, Portsmouth, Hampshire PO6 4BQ

* To whom correspondence should be addressed. Email: stefanie.frank@ucl.ac.uk

1. DNA sequences

Key:

T7 promoter

Ribosome binding site

Gene

Terminator

Strep-tag II

pJL1-sfGFP vector

AGATCAAAGGATCTTCTTGAGATCCTTTTTTTCTGCGCGTAATCTGCTGCTTGCAAACAAAAAAACCACCGCTAC
CAGCGGTGGTTTGTTTGCCGGATCAAGAGCTACCAACTCTTTTTCCGAAGGTAACTGGCTTCAGCAGAGCGCAG
ATACCAAATACTGTTCTTCTAGTGTAGCCGTAGTTAGGCCACCACTTCAAGAACTCTGTAGCACCGCCTACATACC
TCGCTCTGCTAATCCTGTTACCAGTGGCTGCTGCCAGTGGCGATAAGTCGTGTCTTACCGGGTTGGACTCAAGAC
GATAGTTACCGGATAAGGCGCAGCGGTCGGGCTGAACGGGGGGTTCGTGCACACAGCCCAGCTTGGAGCGAA
CGACCTACACCGAACTGAGATACCTACAGCGTGAGCTATGAGAAAGCGCCACGCTTCCCGAAGGGAGAAAGGC
GGACAGGTATCCGGTAAGCGGCAGGGTCGGAACAGGAGAGCGCACGAGGGAGCTTCCAGGGGGAAACGCCT
GGTATCTTTATAGTCCTGTCGGGTTTCGCCACCTCTGACTTGAGCGTCGATTTTTGTGATGCTCGTCAGGGGGGC
GGAGCCTATGGAAAAACGCCAGCAACGCGATCCCGCGAAATTAATACGACTCACTATAGGGAGACCACAACGGT
TTCCCTCTAGAAATAATTTTGTTTAACTTTAAGAAGGAGATATACATATGAGCAAAGGTGAAGAACTGTTTACCGG
CGTTGTGCCGATTCTGGTGGAACTGGATGGCGATGTGAACGGTCACAAATTCAGCGTGCGTGGTGAAGGTGAA
GGCGATGCCACGATTGGCAAACTGACGCTGAAATTTATCTGCACCACCGGCAAACTGCCGGTGCCGTGGCCGAC
GCTGGTGACCACCCTGACCTATGGCGTTCAGTGTTTTAGTCGCTATCCGGATCACATGAAACGTCACGATTTCTTT
AAATCTGCAATGCCGGAAGGCTATGTGCAGGAACGTACGATTAGCTTTAAAGATGATGGCAAATATAAAACGCGC
GCCGTTGTGAAATTTGAAGGCGATACCCTGGTGAACCGCATTGAACTGAAAGGCACGGATTTTAAAGAAGATG
GCAATATCCTGGGCCATAAACTGGAATACAACTTTAATAGCCATAATGTTTATATTACGGCGGATAAACAGAAAAA
TGGCATCAAAGCGAATTTTACCGTTCGCCATAACGTTGAAGATGGCAGTGTGCAGCTGGCAGATCATTATCAGCA
GAATACCCCGATTGGTGATGGTCCGGTGCTGCTGCCGGATAATCATTATCTGAGCACGCAGACCGTTCTGTCTAA

AGATCCGAACGAAAAAGGCACGCGGGACCACATGGTTCTGCACGAATATGTGAATGCGGCAGGTATTACGTGG
AGCCATCCGCAGTTCGAAAAATAAGTCGACCGGCTGCTAACAAAGCCCGAAAGGAAGCTGAGTTGGCTGCTGC
CACCGCTGAGCAATAACTAGCATAACCCCTTGGGGCCTCTAAACGGGTCTTGAGGGGTTTTTTGCTGAAAGCCA
ATTCTGATTAGAAAAACTCATCGAGCATCAAATGAAACTGCAATTTATTCATATCAGGATTATCAATACCATATTTTT
GAAAAAGCCGTTTCTGTAATGAAGGAGAAAACTCACCGAGGCAGTTCCATAGGATGGCAAGATCCTGGTATCGG
TCTGCGATTCCGACTCGTCCAACATCAATACAACCTATTAATTTCCCCTCGTCAAAAATAAGGTTATCAAGTGAGA
AATCACCATGAGTGACGACTGAATCCGGTGAGAATGGCAAAAGCTTATGCATTTCTTTCCAGACTTGTTCAACAG
GCCAGCCATTACGCTCGTCATCAAAATCACTCGCATCAACCAAACCGTTATTCATTCGTGATTGCGCCTGAGCGAG
ACGAAATACGCGATCGCTGTTAAAAGGACAATTACAAACAGGAATCGAATGCAACCGGCGCAGGAACACTGCC
AGCGCATCAACAATATTTTCACCTGAATCAGGATATTCTTCTAATACCTGGAATGCTGTTTTCCCGGGGATCGCAG
TGGTGAGTAACCATGCATCATCAGGAGTACGGATAAAATGCTTGATGGTCGGAAGAGGCATAAATTCCGTCAGCC
AGTTTAGTCTGACCATCTCATCTGTAACATCATTGGCAACGCTACCTTTGCCATGTTTCAGAAACAACTCTGGCGC
ATCGGGCTTCCCATACAATCGATAGATTGTCGCACCTGATTGCCCGACATTATCGCGAGCCCATTTATACCCATATA
AATCAGCATCCATGTTGGAATTTAATCGCGGCTTCGAGCAAGACGTTTCCCGTTGAATATGGCTCATAACACCCCT
TGTATTACTGTTTATGTAAGCAGACAGTTTTATTGTTCATGATGATATATTTTTATCTTGTGCAATGTAACATCAGAG
ATTTTGAGACACAACGTG

AAV5 VP3

ATGTCTGCAGGTGGTGGTGGCCCTCTGGGTGATAATAACCAAGGTGCTGATGGTGTGGGTAACGCGAGCGGTG
ATTGGCACTGCGACAGCACCTGGATGGGCGACCGTGTTGTAACCAAATCTACTCGCACCTGGGTTCTGCCGTCCT
ATAACAACCACCAGTACCGCGAGATTAAAAGCGGTAGCGTTGATGGTAGCAACGCTAACGCTTACTTCGGTTACT
CTACCCCGTGGGGCTACTTCGACTTTAACCGTTTCCATAGCCACTGGTCTCCGCGTGATTGGCAGCGCCTGATCA
ACAACTATTGGGGTTTCCGTCCGCGTTCCCTGCGTGTAAAGATTTTTAACATCCAGGTTAAAGAAGTAACCGTTC
AGGATTCTACTACGACCATTGCCAACAACCTGACTAGCACCGTGCAGGTTTTCACCGATGATGATTACCAGCTGC
CGTACGTGGTCGGTAACGGTACCGAAGGCTGTCTGCCTGCATTCCCGCCACAGGTGTTTACTCTGCCGCAGTACG
GCTATGCCACCCTGAACCGTGACAACACCGAAAACCCGACCGAACGTTCTAGCTTCTTCTGTCTGGAATACTTTC
CTAGCAAAATGCTGCGCACCGGCAACAATTTCGAATTCACCTATAACTTCGAAGAAGTGCCGTTCCACTCTTCCTT
CGCGCCGAGCCAGAACCTGTTCAAACTGGCGAACCCGCTGGTCGATCAGTACCTGTATCGCTTTGTATCTACTAA
CAACACTGGCGGCGTTCAATTTAATAAAAACCTGGCGGGTCGCTACGCAAACACCTATAAAAATTGGTTCCCGG
GTCCGATGGGTCGTACTCAGGGTTGGAACCTGGGCTCTGGTGTTAACCGTGCTTCTGTTAGCGCGTTCGCTACTA
CTAACCGTATGGAACTGGAAGGCGCATCTTACCAGGTACCACCGCAGCCGAACGGTATGACTAACAACCTGCAG
GGCTCTAACACCTATGCGCTGGAAAACACCATGATCTTCAACAGCCAGCCGGCTAACCCGGGTACTACTGCCACG
TATCTGGAGGGTAACATGCTGATCACCTCTGAAAGCGAAACTCAGCCGGTCAACCGCGTTGCTTATAACGTCGGC
GGCCAAATGGCAACTAACAACCAGTCTTCCACCACCGCCCCTGCAACTGGCACCTACAACCTGCAGGAGATCGT
GCCGGGCTCTGTGTGGATGGAACGTGATGTTTACCTGCAGGGCCCGATCTGGGCGAAAATTCCGGAGACCGGT
GCACATTTCCATCCGTCTCCGGCGATGGGTGGTTTCGGTCTGAAACATCCGCCGCCTATGATGCTGATCAAAAAC
ACCCCGGTGCCAGGTAACATCACGTCCTTCTCCGATGTCCCGGTTTCTAGCTTCATTACTCAGTACTCTACCGGTC
AGGTGACCGTGGAAATGGAATGGGAACTGAAAAAAGAAAATAGCAAACGTTGGAACCCGGAAATCCAGTACA
CTAATAACTACAACGACCCGCAATTTGTAGATTTCGCGCCGGATTCTACTGGTGAGTATCGTACCACCCGCCCGAT
CGGTACCCGTTATCTGACGCGTCCGCTGTAA

AAV5 VP3 C-terminal Strep-tag II

ATGTCTGCAGGTGGTGGTGGCCCTCTGGGTGATAATAACCAAGGTGCTGATGGTGTGGGTAACGCGAGCGGTG
ATTGGCACTGCGACAGCACCTGGATGGGCGACCGTGTTGTAACCAAATCTACTCGCACCTGGGTTCTGCCGTCCT
ATAACAACCACCAGTACCGCGAGATTAAAAGCGGTAGCGTTGATGGTAGCAACGCTAACGCTTACTTCGGTTACT
CTACCCCGTGGGGCTACTTCGACTTTAACCGTTTCCATAGCCACTGGTCTCCGCGTGATTGGCAGCGCCTGATCA
ACAACTATTGGGGTTTCCGTCCGCGTTCCCTGCGTGTAAAGATTTTTAACATCCAGGTTAAAGAAGTAACCGTTC
AGGATTCTACTACGACCATTGCCAACAACCTGACTAGCACCGTGCAGGTTTTCACCGATGATGATTACCAGCTGC

CGTACGTGGTCGGTAACGGTACCGAAGGCTGTCTGCCTGCATTCCCGCCACAGGTGTTTACTCTGCCGCAGTACG
GCTATGCCACCCTGAACCGTGACAACACCGAAAACCCGACCGAACGTTCTAGCTTCTTCTGTCTGGAATACTTTC
CTAGCAAAATGCTGCGCACCGGCAACAATTTCGAATTCACCTATAACTTCGAAGAAGTGCCGTTCCACTCTTCCTT
CGCGCCGAGCCAGAACCTGTTCAAACTGGCGAACCCGCTGGTCGATCAGTACCTGTATCGCTTTGTATCTACTAA
CAACACTGGCGGCGTTCAATTTAATAAAAACCTGGCGGGTCGCTACGCAAACACCTATAAAAATTGGTTCCCGG
GTCCGATGGGTCGTACTCAGGGTTGGAACCTGGGCTCTGGTGTTAACCGTGCTTCTGTTAGCGCGTTCGCTACTA
CTAACCGTATGGAACTGGAAGGCGCATCTTACCAGGTACCACCGCAGCCGAACGGTATGACTAACAACCTGCAG
GGCTCTAACACCTATGCGCTGGAAAACACCATGATCTTCAACAGCCAGCCGGCTAACCCGGGTACTACTGCCACG
TATCTGGAGGGTAACATGCTGATCACCTCTGAAAGCGAAACTCAGCCGGTCAACCGCGTTGCTTATAACGTCGGC
GGCCAAATGGCAACTAACAACCAGTCTTCCACCACCGCCCCTGCAACTGGCACCTACAACCTGCAGGAGATCGT
GCCGGGCTCTGTGTGGATGGAACGTGATGTTTACCTGCAGGGCCCGATCTGGGCGAAAATTCCGGAGACCGGT
GCACATTTCCATCCGTCTCCGGCGATGGGTGGTTTCGGTCTGAAACATCCGCCGCCTATGATGCTGATCAAAAAC
ACCCCGGTGCCAGGTAACATCACGTCCTTCTCCGATGTCCCGGTTTCTAGCTTCATTACTCAGTACTCTACCGGTC
AGGTGACCGTGGAAATGGAATGGGAACTGAAAAAAGAAAATAGCAAACGTTGGAACCCGGAAATCCAGTACA
CTAATAACTACAACGACCCGCAATTTGTAGATTTCGCGCCGGATTCTACTGGTGAGTATCGTACCACCCGCCCGAT
CGGTACCCGTTATCTGACGCGTCCGCTGTGGAGCCATCCGCAGTTCGAAAAATAA

AAV5 VP3 N-terminal Strep-tag II

ATGTGGAGCCATCCGCAGTTCGAAAAAATGTCTGCAGGTGGTGGTGGCCCTCTGGGTGATAATAACCAAGGTGC
TGATGGTGTGGGTAACGCGAGCGGTGATTGGCACTGCGACAGCACCTGGATGGGCGACCGTGTTGTAACCAAA
TCTACTCGCACCTGGGTTCTGCCGTCCTATAACAACCACCAGTACCGCGAGATTAAAAGCGGTAGCGTTGATGGT
AGCAACGCTAACGCTTACTTCGGTTACTCTACCCCGTGGGGCTACTTCGACTTTAACCGTTTCCATAGCCACTGGT
CTCCGCGTGATTGGCAGCGCCTGATCAACAACTATTGGGGTTTCCGTCCGCGTTCCCTGCGTGTAAAGATTTTTA
ACATCCAGGTTAAAGAAGTAACCGTTCAGGATTCTACTACGACCATTGCCAACAACCTGACTAGCACCGTGCAGG
TTTTCACCGATGATGATTACCAGCTGCCGTACGTGGTCGGTAACGGTACCGAAGGCTGTCTGCCTGCATTCCCGC
CACAGGTGTTTACTCTGCCGCAGTACGGCTATGCCACCCTGAACCGTGACAACACCGAAAACCCGACCGAACGT
TCTAGCTTCTTCTGTCTGGAATACTTTCCTAGCAAAATGCTGCGCACCGGCAACAATTTCGAATTCACCTATAACTT
CGAAGAAGTGCCGTTCCACTCTTCCTTCGCGCCGAGCCAGAACCTGTTCAAACTGGCGAACCCGCTGGTCGATC
AGTACCTGTATCGCTTTGTATCTACTAACAACACTGGCGGCGTTCAATTTAATAAAAACCTGGCGGGTCGCTACGC
AAACACCTATAAAAATTGGTTCCCGGGTCCGATGGGTCGTACTCAGGGTTGGAACCTGGGCTCTGGTGTTAACC
GTGCTTCTGTTAGCGCGTTCGCTACTACTAACCGTATGGAACTGGAAGGCGCATCTTACCAGGTACCACCGCAGC
CGAACGGTATGACTAACAACCTGCAGGGCTCTAACACCTATGCGCTGGAAAACACCATGATCTTCAACAGCCAG
CCGGCTAACCCGGGTACTACTGCCACGTATCTGGAGGGTAACATGCTGATCACCTCTGAAAGCGAAACTCAGCC
GGTCAACCGCGTTGCTTATAACGTCGGCGGCCAAATGGCAACTAACAACCAGTCTTCCACCACCGCCCCTGCAA
CTGGCACCTACAACCTGCAGGAGATCGTGCCGGGCTCTGTGTGGATGGAACGTGATGTTTACCTGCAGGGCCCG
ATCTGGGCGAAAATTCCGGAGACCGGTGCACATTTCCATCCGTCTCCGGCGATGGGTGGTTTCGGTCTGAAACA
TCCGCCGCCTATGATGCTGATCAAAAACACCCCGGTGCCAGGTAACATCACGTCCTTCTCCGATGTCCCGGTTTCT
AGCTTCATTACTCAGTACTCTACCGGTCAGGTGACCGTGGAAATGGAATGGGAACTGAAAAAAGAAAATAGCAA
ACGTTGGAACCCGGAAATCCAGTACACTAATAACTACAACGACCCGCAATTTGTAGATTTCGCGCCGGATTCTAC
TGGTGAGTATCGTACCACCCGCCCGATCGGTACCCGTTATCTGACGCGTCCGCTGTAA

2. AAV5 VP3 full western blots

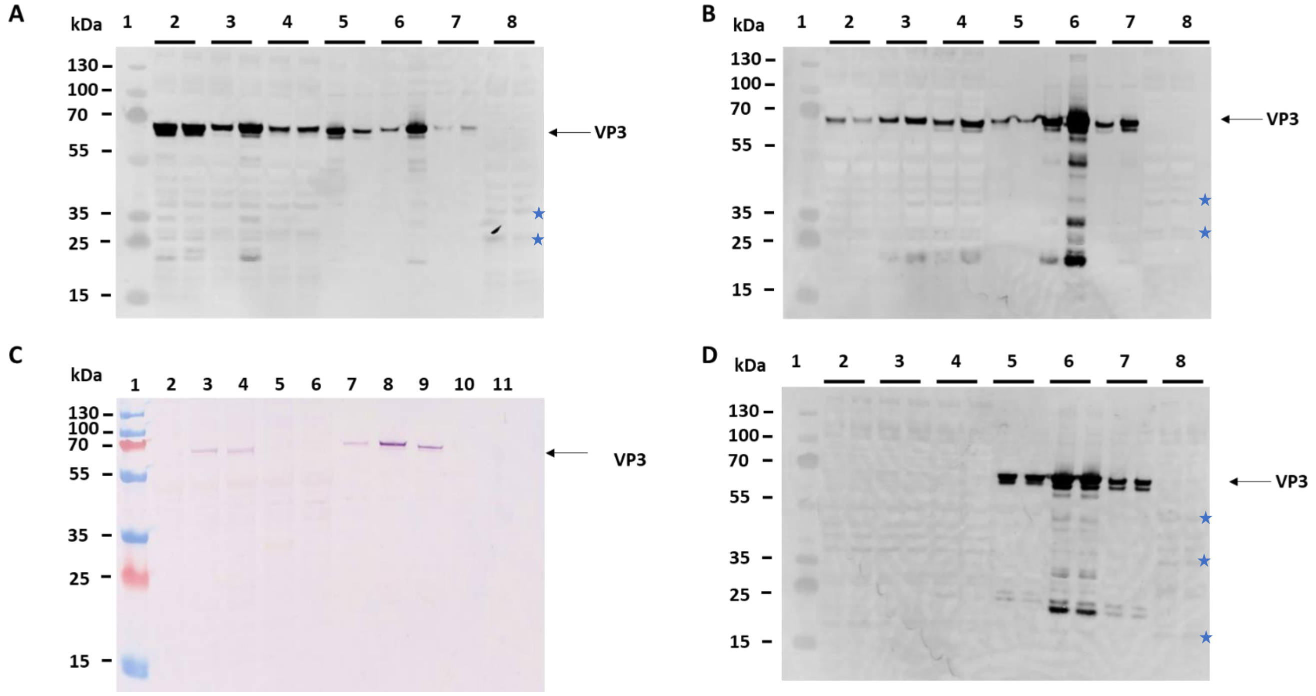

Figure S1. Western blots of AAV5 VP3 samples. A)=18°C, B)=24°C, C)=30°C and D)=37°C. Blue stars indicate non-specific bands from a no-plasmid control. A) B) D) are ordered as follows, lane 1=molecular ladder, lane 2=AAV5 VP3 N-terminal Strep-tag II soluble fraction, lane 3=AAV5 VP3 N-terminal Strep-tag II insoluble fraction, lane 4=AAV5 VP3 soluble fraction, lane 5=AAV5 VP3 N-terminal Strep-tag II insoluble fraction, lane 6=AAV5 VP3 C-terminal Strep-tag II insoluble fraction, lane 7=AAV5 VP3 insoluble fraction, lane 8=no plasmid control. Where there are two samples represented by one lane these are biological repeats. C) Lane 1=molecular weight ladder, lane 2=AAV5 VP3 N-terminal Strep-tag II soluble fraction, lane 3=AAV5 VP3 C-terminal Strep-tag II soluble fraction, lane 4=AAV5 VP3 soluble fraction, lane 5=pJL1-sfGFP CFPS reaction soluble control, lane 6=no plasmid control soluble fraction, lane 7=AAV5 VP3 N-terminal Strep-tag II insoluble fraction, lane 8=AAV5 VP3 C-terminal Strep-tag II insoluble fraction, lane 9=AAV5 VP3 insoluble fraction, lane 10= pJL1-sfGFP CFPS reaction insoluble control, lane 11=no plasmid control insoluble fraction.

3. **Strep-Tactin® western blot of purification products**

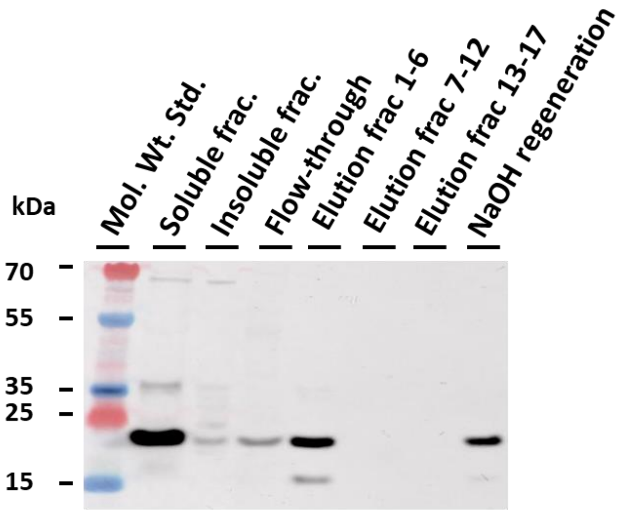

Figure S2. Strep-Tactin®-HRP (BioRad) western blot of N-terminal Strep-tag II AAV5 VP3 purification process using StrepTrapXT™ column.

4. ELISA standard curve

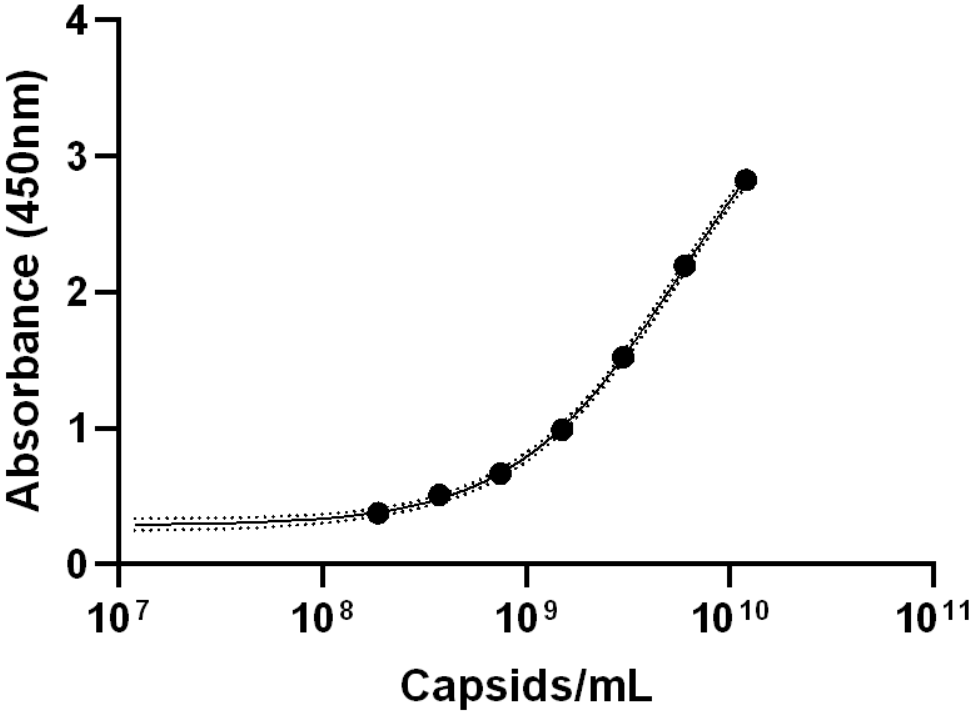

Figure S3. Standards from AAV5 Xpress ELISA (Progen) plotted into a log10 curve using a four parameter logistic regression model. The R2 value is equal to 0.99. Values were obtained from kit standards run in duplicate. The value interpolated off this graph to determine a capsid titre for purified AAV5 VP3 N-terminal Strep-tag II was 0.67±0.03 (AU).

5. DLS analysis by volume and number

Since DLS analysis favours large particles as they have increased scattering intensity results can often looked skewed to seem like there are more large particles in a sample than is accurate. Therefore results normalised to take into account number of particles and volume can offer results more representative of the sample. These distributions are based on the intensity scatter plot and redistributed by mathematical equation.

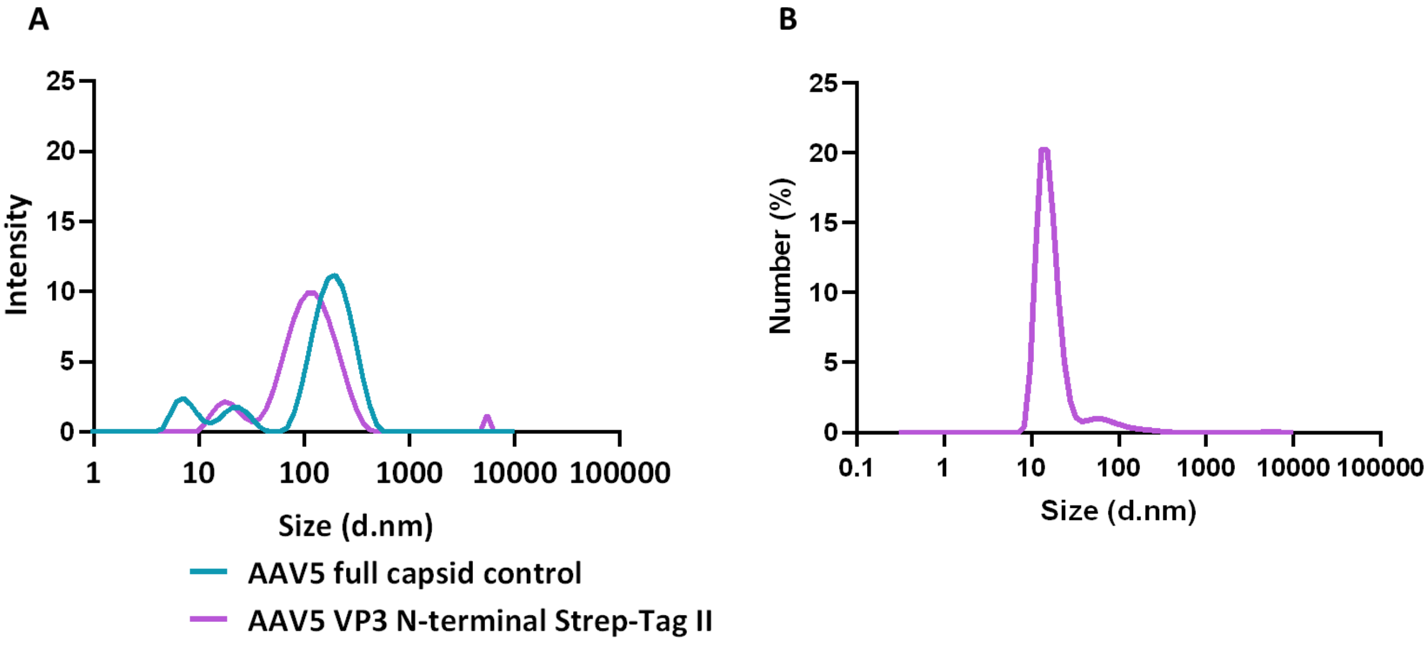

Figure S4. DLS plots A) shows original DLS plot measured by intensity. From left to right, the full capsid control (empty AAV5 particles, Progen) peaks at 7.10 d. nm, 23.8 d. nm and 197 d. nm. From left to right, AAV5 VP3 peaks at 20.23 d.nm, 108 d.nm and 5468 d.dm. Each line is an automatically generated average of 3 reads. B) AAV5 VP3 N-terminal Strep-tag II DLS read normalised by number from A) peaking at 15.1 d. nm.

6. Representative TEM image

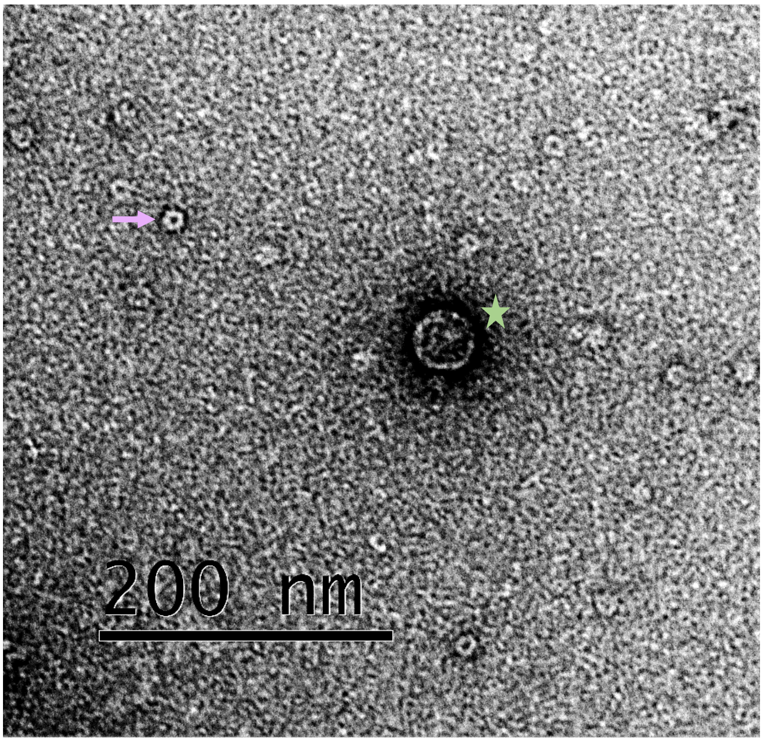

Figure S5. TEM image of AAV5 VP3 N-terminal Strep-tag II (purple arrow) with inclusion of unknown large contaminants (green star).
